# PARALLEL MEASUREMENTS OF PROTEIN AND CELL TURNOVER REVEAL HOW TISSUE CONTEXT AND AGING SHAPE PROTEIN LIFETIMES

**DOI:** 10.1093/geroni/igac059.1667

**Published:** 2022-12-20

**Authors:** Abigail Buchwalter, Kevin Welle, Jennifer Hryhorenko, Sina Ghaemmaghami, John Hasper

**Affiliations:** University of California, San Francisco, San Francisco, California, United States; Mass Spectrometry Resource Lab, University of Rochester, Rochester, New York, United States; Mass Spectrometry Resource Laboratory, University of Rochester, Rochester, California, United States; Department of Biology, University of Rochester, Rochester, New York, United States; Cardiovascular Research Institute, University of California, San Francisco, San Francisco, California, United States

## Abstract

The lifespans of proteins can range from moments to years within mammalian tissues. Protein lifespan is relevant to organismal aging, as long-lived proteins can accrue damage over time. It is unclear how protein lifetime is shaped by tissue context, where both cell division and proteolytic degradation contribute to protein turnover. We have developed turnover and replication analysis by 15N isotope labeling (TRAIL) for parallel quantification of protein and cell lifetimes. We have deployed TRAIL over 32 days in 4 mouse tissues to date to quantify cell proliferation with high precision and no toxicity and determine that protein lifespan varies independently of cell lifespan. Variation in protein lifetime is non-random: multiprotein complexes such as the ribosome have consistent lifetimes across tissues, while mitochondria, peroxisomes, and lipid droplets have variable lifetimes across tissues. To model the effects of aging on tissue homeostasis, we apply TRAIL to a mouse model of Hutchinson-Gilford progeria syndrome and uncover fat-specific alterations in cell lifetime and proteome composition, as well as a broad decrease in protein turnover flux. These data indicate that environmental factors influence protein turnover in vivo and provide a framework to understand proteome aging in tissue context.

